# Tangential Flow Ultrafiltration Allows Purification and Concentration of Lauric Acid-/Albumin-Coated Particles for Improved Magnetic Treatment

**DOI:** 10.3390/ijms160819291

**Published:** 2015-08-14

**Authors:** Jan Zaloga, Marcus Stapf, Johannes Nowak, Marina Pöttler, Ralf P. Friedrich, Rainer Tietze, Stefan Lyer, Geoffrey Lee, Stefan Odenbach, Ingrid Hilger, Christoph Alexiou

**Affiliations:** 1 Section of Experimental Oncology and Nanomedicine (SEON), Else Kröner-Fresenius Stiftungsprofessur for Nanomedicine, University Hospital Erlangen, 91054 Erlangen, Germany; E-Mails: jan.zaloga@uk-erlangen.de (J.Z.); marina.poettler@uk-erlangen.de (M.P.); ralf.friedrich@uk-erlangen.de (R.P.F.); rainer.tietze@uk-erlangen.de (R.T.); stefan.lyer@uk-erlangen.de (S.L.); 2 Institute for Diagnostic and Interventional Radiology, University Hospital Jena, 07747 Jena, Germany; E-Mails: Marcus.Stapf@med.uni-jena.de (M.S.), Ingrid.Hilger@med.uni-jena.de (I.H.); 3 Chair of Magnetofluiddynamics, Measuring and Automation Technology, Technische Universität Dresden, 01069 Dresden, Germany; E-Mails: johannes.nowak@tu-dresden.de (J.N.); stefan.odenbach@tu-dresden.de (S.O.); 4 Division of Pharmaceutics, Friedrich Alexander University Erlangen-Nuremberg, 91058 Erlangen, Germany; E-Mail: geoff.lee@fau.de

**Keywords:** hyperthermia, nanoparticle concentration, tangential ultrafiltration, nanoparticle purification, specific absorption rate (SAR), superparamagnetic iron oxide nanoparticles (SPIONs)

## Abstract

Superparamagnetic iron oxide nanoparticles (SPIONs) are frequently used for drug targeting, hyperthermia and other biomedical purposes. Recently, we have reported the synthesis of lauric acid-/albumin-coated iron oxide nanoparticles SEON^LA-BSA^, which were synthesized using excess albumin. For optimization of magnetic treatment applications, SPION suspensions need to be purified of excess surfactant and concentrated. Conventional methods for the purification and concentration of such ferrofluids often involve high shear stress and low purification rates for macromolecules, like albumin. In this work, removal of albumin by low shear stress tangential ultrafiltration and its influence on SEON^LA-BSA^ particles was studied. Hydrodynamic size, surface properties and, consequently, colloidal stability of the nanoparticles remained unchanged by filtration or concentration up to four-fold (*v*/*v*). Thereby, the saturation magnetization of the suspension can be increased from 446.5 A/m up to 1667.9 A/m. *In vitro* analysis revealed that cellular uptake of SEON^LA-BSA^ changed only marginally. The specific absorption rate (SAR) was not greatly affected by concentration. In contrast, the maximum temperature *T*_max_ in magnetic hyperthermia is greatly enhanced from 44.4 °C up to 64.9 °C by the concentration of the particles up to 16.9 mg/mL total iron. Taken together, tangential ultrafiltration is feasible for purifying and concentrating complex hybrid coated SPION suspensions without negatively influencing specific particle characteristics. This enhances their potential for magnetic treatment.

## 1. Introduction

Superparamagnetic iron oxide nanoparticles (SPIONs) are frequently used for magnetic drug targeting [1,2], magnetic hyperthermia [3,4,5] and other purposes, like imaging [6]. For these applications, high iron concentrations, biocompatibility, colloidal stability and relatively low viscosity of the suspension are of great importance [7,8,9,10]. Recently we have reported the synthesis of lauric acid/albumin hybrid-coated SPIONs for magnetic drug targeting called SEON^LA-BSA^. These particles exhibited very promising properties for magnetic drug targeting applications, which were derived from the nature of the hybrid coating. This coating consists of fatty acids, which act as anchoring groups on the surface, to which albumin is chemisorbed. Free albumin remained in the sample despite repeated ultrafiltration using conventional centrifuge ultrafiltration units, as the molecular weight cut-off (MWCO) was limited by the particle size [11]. However, removal of free albumin is desirable in this case, as drugs can also bind to free albumin, which is not located on the particle surface [12]. This would exclude those drug molecules from magnetic attraction and could cause side effects. Furthermore, excess protein can potentially increase the viscosity of the samples. In this regard, it has been shown earlier that the viscosity, which could even be influenced by an external magnetic field as such [13], might strongly effect the efficiency of drug targeting, or magnetic hyperthermia [14], or particle attraction in magnetic drug targeting [15]. Conventional methods, like centrifugal ultrafiltration, for the purification and concentration of nanoparticle suspensions are not always convenient [16], as they often involve high shear stress and are time consuming and expensive. The purification rate for macromolecules like albumin can also be low at appropriate molecular weight cut-offs, as the sizes of individual particles and albumin molecules are very similar. Although higher cut-offs can improve the protein removal rate, iron oxide particles can penetrate the membrane at high centrifugal forces during diafiltration, too. Furthermore, the high shear stress necessary for this filtration can promote the aggregation of lyophobic colloids. Here, tangential flow ultrafiltration has been reported to provide a useful alternative [17]. Its main difference to dead-end methods like diafiltration is that the substance is tangentially passed across a filtration membrane with relatively low pressure. Thereby, tangential flow ultrafiltration avoids the formation of aggregates and local high concentrations by high centrifugal forces. This was also reported to lead to much higher filtration efficiency [16].

We studied the removal of excess protein and its influence on the particle characteristics using tangential ultrafiltration. The removal of stabilizing agents on colloidally-suspended particles may dramatically influence the colloid’s characteristics, as it can enhance aggregation and instability [18,19]. We therefore investigated the influence of protein removal and particle concentration on the hydrodynamic cluster size of the particles. Colloidal stability in whole blood is a necessary condition for the feasibility of a ferrofluid for use intravasally [20]. Investigating pH-dependent electrophoretic mobility and size measurements as proposed by Tombacz *et al.* [21], we proved that the surface of the nanoparticles was not affected by the filtration and that only free albumin was removed from the sample.

Enhancing the iron concentration of aqueous SPION suspensions is preferable for many biomedical applications. Therefore, we investigated the possibility of concentrating SEON^LA-BSA^ suspensions using tangential ultrafiltration while monitoring the hydrodynamic cluster size and colloidal stability. As the attractability of magnetic particles depends on the magnetic susceptibility of the suspension as such [15], it is desirable to increase the latter to achieve better enrichment of particles in magnetic drug targeting. Higher concentrations may enable better enrichment of SPIONs and, therefore, better local enrichment of drugs.

Protein coatings or coronas are known to affect the cellular uptake of nanoparticles in general and iron oxide nanoparticles in particular [22,23]. Using an established colorimetric method [24], we investigated cellular uptake of SEON^LA-BSA^ into human T-lymphoma cells before and after filtration.

In order to demonstrate the influence of filtration and particle concentration on the feasibility of particles for magnetic hyperthermia, we determined the specific absorption rate (SAR) [25]. In addition to the SAR measurements, the maximal achievable heating temperature (*T*_max_) was determined. Increased temperatures can increase the cytotoxic efficiency of several anticancer drugs, including mitoxantrone [26], which has been the drug of choice of our previous studies, both *in vitro* [11] and *in vivo* [27,28]. Furthermore, magnetic hyperthermia treatment alone can induce cytotoxic effects on tumor cells when appropriate temperatures above 41‒42 °C are achieved [29]. Higher temperatures of 50 °C and above can lead to thermoablation, which is much more effective, but on the other hand, bears the risk of the burst release of toxins by tumor necrosis [5]. Another very important questions is that the surrounding medium in tumor tissues might lower the heating capacity of particles [9]. Thus, it seems desirable to have the possibility to achieve *T*_max_ values as possible *in vitro* and then to carefully monitor the therapeutic outcome depending on the magnetic field conditions *in vivo*. We believe that the presented data can conclusively demonstrate the feasibility of tangential flow ultrafiltration for the purification and concentration of macromolecule-coated SPIONs like SEON^LA-BSA^ and its feasibility for the optimization of magnetic treatment.

## 2. Results and Discussion

### 2.1. Filtration and Concentration Efficiency

**Figure 1:**
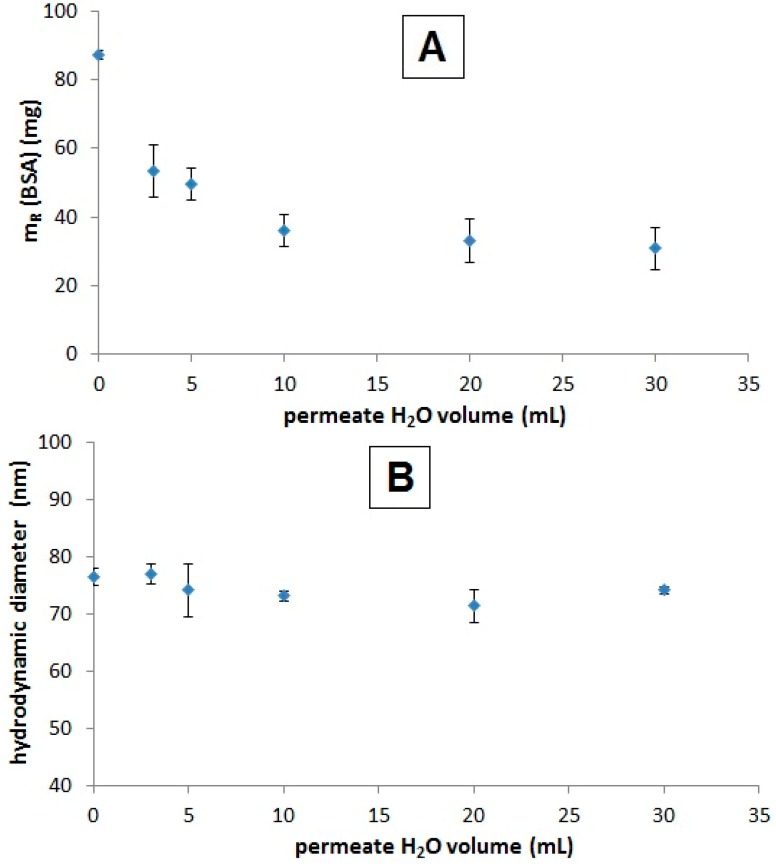
Effect of ultrafiltration on protein content and hydrodynamic aggregate size of SEON^LA-BSA^. The relative dry mass (**A**) of SEON^LA-BSA^ (V = 5 mL) was calculated after ultrafiltration with different volumes of ultrapure water as the washing agent. The mass displayed is relative to the mass of the precursor SEON^LA^ in order to discriminate the mass of albumin from the mass of the particle cores; (**B**) Photon cross-correlation spectroscopy (PCCS) measurements displayed as volume mean diameter of the samples from (**A**). All measurements were performed in triplicate.

Drying loss experiments proved that excess protein is rapidly removed from the suspension by ultrafiltration, which is shown in Figure 1. The relative amount of removed protein from 5 mL of SEON^LA-BSA^ reaches a plateau after washing with at least a double excess of H_2_O. This equals 3% (m/V) of BSA remaining in suspension, which was reported earlier to be the minimal concentration required to achieve successful synthesis [11]. While the protein is removed from the surface, no distinct changes in hydrodynamic cluster sizes were observed.

**Figure 2:**
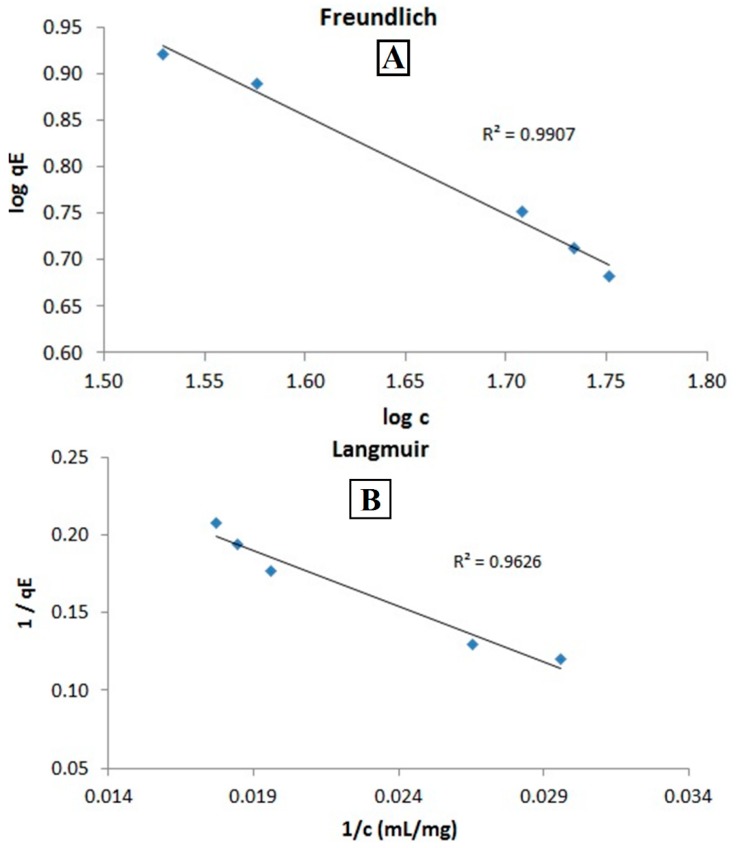
Linear plots of desorption isotherms of albumin from SEON^LA-BSA^. Freundlich (**A**) and Langmuir (**B**) isotherms showed a good determination coefficient *R*^2^. Apparently, the Freundlich model was able to better describe the desorption of albumin from the particle matrix with an *R*^2^ of 0.9907 and a slope of 1/*n* = −1.06.

As shown in Figure 2, the Freundlich isotherm is able to describe the desorption process with a coefficient of determination (*R*^2^) of 0.9907, whereas the Langmuir isotherm, which does not include interactions between different sorbent molecules, fits with an *R*^2^ of only 0.9626. Indeed, a modulus of 1/*n* = 1.06 in the Freundlich isotherm indicates close to linear sorption. At constant p and T, this means that the sorption energies for the desorption sites are very similar or close to equal. This may indicate the very weak interaction of excess albumin molecules to the primary layers on the particle surfaces, which is supportive of the matrix-like structure of the protein excess we reported earlier [11]. This interaction could derive from electrostatic interaction according to Debye–Hückel theory.

**Figure 3:**
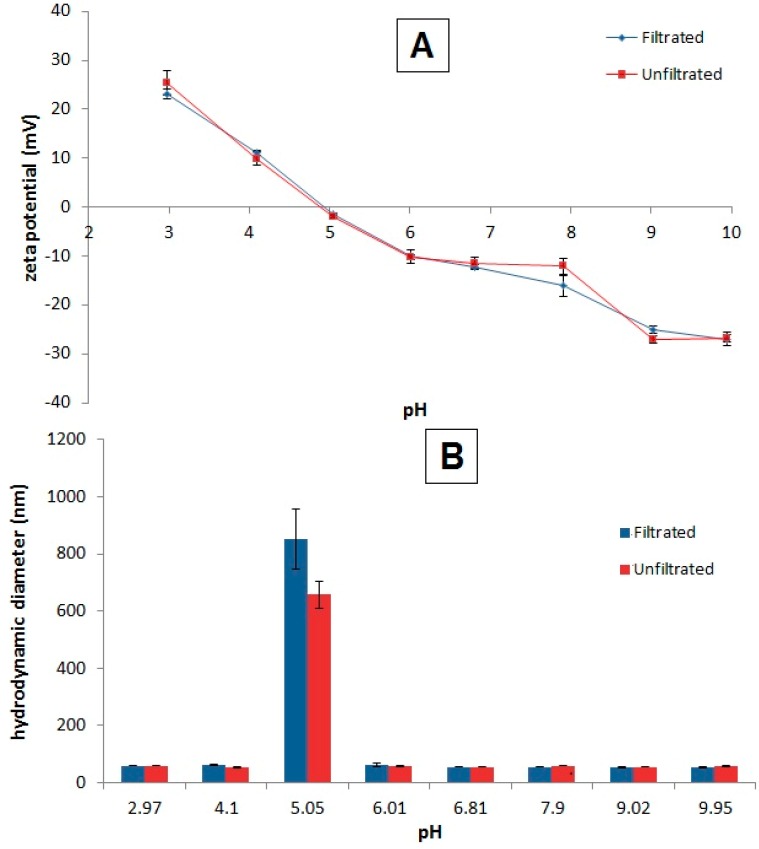
pH-dependent zeta potential and hydrodynamic size measurements. The development of (**A**) the zeta potential and (**B**) the hydrodynamic diameter (z-average) depending on the pH as measured by DLS. SEON^LA-BSA^ before filtration is depicted in red; SEON^LA-BSA^ after filtration with a double excess volume of ultrapure water is depicted in blue. All measurements were performed in triplicate 30 s after sonication.

The pH-dependent analysis of hydrodynamic size and zeta potential enable a comparison of the surface properties of particles [21]. Depending on the chemical composition of the particle surface, the pH-dependence of the surface charge, which in the case of electrostatic stabilization strongly correlates with the hydrodynamic size, is altered. Figure 3 shows the clear pH dependence of the surface charge of SEON^LA-BSA^ before and after tangential flow ultrafiltration. The results prove that in both cases, the point of zero charge (PZC) of the particles lies at approximately pH 5. This indicates that the outer layer on the particle surface consisted of albumin, the isoelectric point of which lies at the same pH [30]. In the case of the original SEON^LA-BSA^ particles, this is consistent with previous results [11]. The PZC of SEON^LA-BSA^ was not changed after filtration, which proves that the surface properties did not change. The removed protein was indeed only excess protein, and the albumin that was adsorbed to the particle surface using the strong interaction with the fatty acid monolayer was not removed. This is further supportive of the core-shell structure of SEON^LA-BSA^, which was proposed earlier [11]. The loss of surface charge at the PZC correlated with an increase in hydrodynamic size, albeit without the formation of visible precipitates. This indicates that the stabilization mechanism of the suspension is mainly derived from electrostatic repulsion in both cases. However, the results show that at physiological pH, the electrostatic repulsion of SEON^LA-BSA^ particles is sufficient to retain the colloidal stability of the ferrofluid.

**Figure 4:**
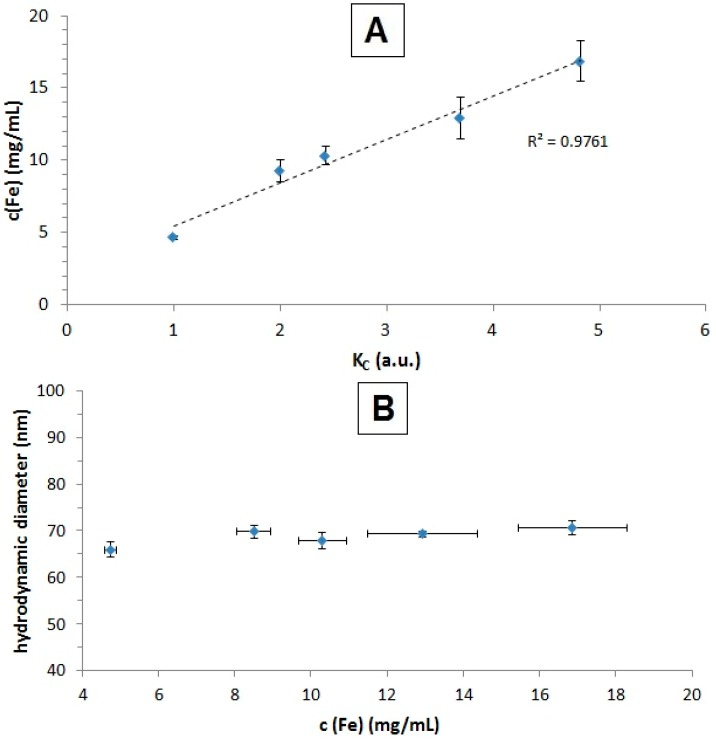
Efficiency of the concentration and the effect on the hydrodynamic aggregate size of SEON^LA-BSA^. The iron content of SEON^LA-BSA^ (**A**) depending on the ratio of the starting volume/end volume (concentration factor); (**B**) PCCS measurements of the hydrodynamic diameters of the samples from (**A**). All measurements for (**A**,**B**) were performed in triplicate.

Using air instead of water as the pressure balance, we were able to concentrate the samples. After washing with 20 mL of ultrapure water in order to remove excess protein before concentration, we concentrated the sample up to four-fold (*v*/*v*). As shown in Figure 4, the iron concentration can be increased from 4.65 ± 0.14 mg/mL up to 16.9 ± 1.43 mg/mL, linear to the concentration rate K_c_ (starting volume/end volume). This does not significantly (*p* > 0.05) affect the hydrodynamic aggregate size. This also means that no loss of particles, even at higher concentrations, had occurred. This is in concordance with the chosen filter cut-off, which was 100 kDa, which equals a pore size of around 10 nm [31]. The size distribution of SEON^LA-BSA^ before and after filtration, which is displayed in Supplementary Figure S1, shows no changes in the curve shape. Furthermore, it shows that the lower particle cluster size starts at around 15 nm, which is just above the pore size.

The robustness and reliability of the UV-VIS method that was used in this study is comparable to sophisticated methods, like microwave plasma-assisted atom emission spectroscopy (MP-AES), in these given conditions. Measurements of the same samples using the same sample preparation method (Supplementary Figure S2) showed no statistically significant differences between the values.

### 2.2. Effect of Filtration on Magnetic Properties of SEON^LA-BSA^

**Figure 5:**
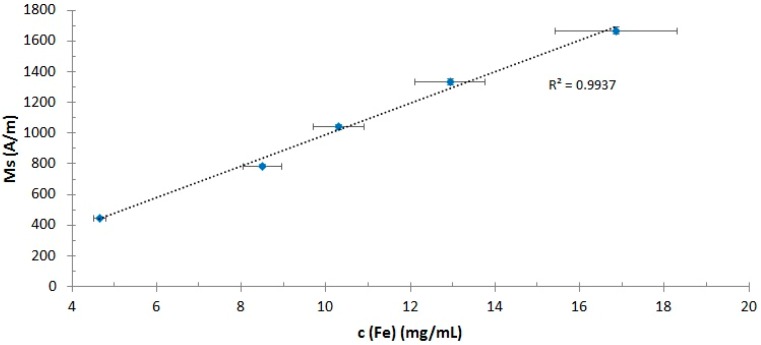
Increase of the saturation magnetization of SEON^LA-BSA^ of the samples. The saturation magnetization increases in a linear way (*R*^2^ = 0.9937) with the respective iron content of the samples, which was measured in triplicate using the aforementioned UV-VIS method.

As expected from previous results [11], the magnetic fluids displayed superparamagnetic behavior, as none of the samples showed remanence magnetization. In Figure 5, the linear dependency of the saturation magnetization M_S_ of SEON^LA-BSA^ on the iron concentration of the sample is shown. The original M_S_ of the unconcentrated sample (446.5 A/m) was increased over 3.7-fold to 1667.9 A/m for the highest concentrated sample. This enhances the feasibility of SEON^LA-BSA^ for magnetism-based therapies, as increasing iron concentrations could enable higher enrichment in magnetic drug targeting [15] and higher maximum achievable temperatures *T*_max_ during magnetic hyperthermia. As there is no alternation of the magnetic behavior of the SPIONs, the effects due to particle-particle interaction at higher concentrations, like aggregation effects, are not occurring in this case. The specific magnetization, which is calculated by dividing M_S_ by the volume fraction of magnetic material Φ, lies at 450.2 ± 6.8, which is in good concordance with previous results [11].

### 2.3. Effect of Filtration on Colloidal Stability and Cellular Uptake

**Figure 6:**
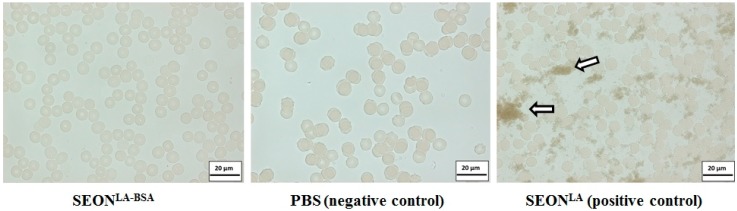
Blood stability assays. Exemplary light microscopy images of (from **left** to **right**) concentrated SEON^LA-BSA^ (c(Fe) = 16.9 ± 1.43 mg/mL), PBS and SEON^LA^ (c(Fe) = 17.2 ± 0.69 mg/mL) diluted 1:2 in whole blood stabilized with EDTA. The arrows mark exemplary particle aggregates.

Even the highest concentrated sample of SEON^LA-BSA^, which exhibited a total iron concentration of 16.9 ± 1.43 mg/mL, was macroscopically stable in EDTA-stabilized whole blood over an observation time of 24 h. Aggregates with sizes above 300 nm, which is the resolution limit of the microscope, were not observed in the microscopy images (Figure 6). However, the image was darkened by the presence of the SPIONs. Complimentary PCCS measurement of the same sample in RPMI 1640 cell culture media showed a volume mean diameter of 62.42 ± 1.75 nm, which proves that the sample is colloidally stable against complex biological fluids. This proves that SEON^LA-BSA^ is highly stable in blood, even in the presence of iron-chelating agents, like EDTA. This effect is essential for use intravasally [20].

**Figure 7:**
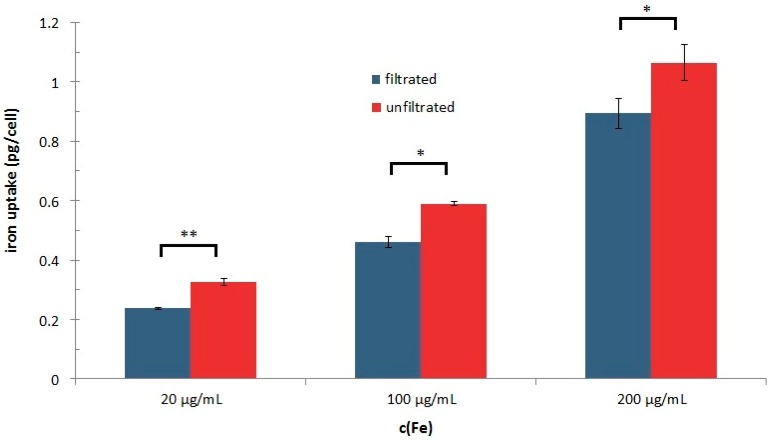
Influence of tangential ultrafiltration on cellular uptake of SEON^LA-BSA^. Total iron oxide concentration of filtrated (blue bars) and un-filtrated (red bars) SEON^LA-BSA^ in Jurkat cells after 48 h of incubation. The uptake was significantly reduced by filtration for all three concentrations tested (* *p* < 0.05; ** *p* < 0.005). All measurements were performed in triplicate; the background was subtracted.

The biocompatibility of SEON^LA-BSA^ has been studied extensively *in vitro* before. After tangential ultrafiltration, the uptake of SEON^LA-BSA^ into Jurkat cells was significantly reduced, as shown in Figure 7 (*p* < 0.05). At total iron concentrations of 100 µg/mL, the average uptake of iron into a single cell was measured at 0.479 ± 0.051 pg for filtrated particles, whereas the amount for un-filtrated particles was 0.608 ± 0.061 pg per cell. This is quite surprising, as proteins are known to reduce the uptake of particles [23]. However, our results show that excess protein is not necessarily located at the direct surface of the particles and might increase endocytosis of the cells by itself, thereby possibly enhancing particle uptake.

### 2.4. Dependence of SAR and T_max_ on Filtration and Iron Oxide Concentration

**Figure 8:**
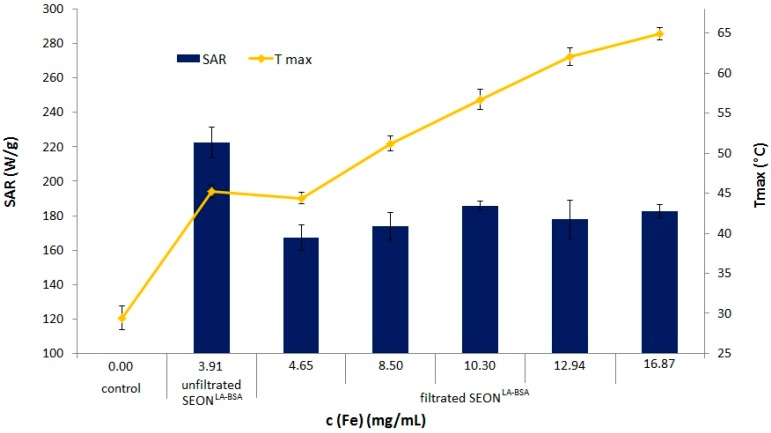
SAR and *T*_max_ of SEON^LA-BSA^ after filtration and/or concentration. Specific absorption rates (blue bars) and maximum heating temperatures (yellow dots) of un-filtrated SEON^LA-BSA^ and filtrated and concentrated SEON^LA-BSA^. All measurements were performed in triplicate.

As can be seen in Figure 8, the SAR is significantly (*p* < 0.005) reduced by 21.8% after removal of excess protein. This observed reduction can only very unlikely be attributed to thermal losses, since the same experimental setup with an isolator to exclude external influences was used. Additionally, the measurement of the temperature was performed centrally inside the ferrofluid in a closed system to minimize the effects of thermal losses. As the hydrodynamic cluster size is not affected by the addition or removal of albumin, the effect must be related to matrix effects deriving from the protein. As the desorption curve indicates a non-linear desorption under approximately constant filtration conditions, this could be indicating that the protein matrix is affecting relaxation after magnetization in the alternating field. 

As expected, the concentration of the particles did not affect the SAR. The heating capacity of the ferrofluid per volume was increasing in accordance to the total iron concentration. The highest achievable temperature for these particles under the given alternating magnetic field (AMF) parameters is 64.9 °C, equaling to a total increase of 46.2%, which is achievable by the chosen filtration conditions. The presented results clearly show that the described filtration method is able to concentrate magnetic particles without negatively influencing the SAR. Hence, higher maximal temperatures are achievable by the concentration of ferrofluids, allowing huge flexibility for hyperthermal [29] or even thermoablative treatments [5]. Additionally, combination therapies can be further optimized, since the SPION-drug relation can be adapted by a subsequent consistent target temperature in the tumor tissue.

## 3. Experimental Section

### 3.1. Materials and Chemicals

Iron (II) chloride tetrahydrate (FeCl_2_·4H_2_O), hydroxylammonium chloride and bovine serum albumin (BSA) were purchased from Merck (Darmstadt, Germany). Iron (III) chloride hexahydrate (FeCl_3_·6H_2_O), dialysis tubes (Spectrapor 6, MWCO 8 kDa), ammonium chloride, sodium hydroxide, sodium chloride, formic acid, hydrochloric acid 25%, sodium dodecyl sulfate (SDS) and ammonia solution 25% were supplied by Roth (Karlsruhe, Germany). Phosphate-buffered saline (PBS), ammonium formate, lauric acid and acetone were purchased from Sigma-Aldrich (St. Louis, MO, USA). Sulfosalicylic acid solution 20% was bought from Applichem (Darmstadt, Germany). Ringer’s solution was bought from Baxter Healthcare (Zurich, Switzerland). Falcon ultrafiltration tubes (MWCO 100 kDa) were purchased from Sartorius (Gottingen, Germany).

### 3.2. Nanoparticles and Filtration

SPION particles with a hybrid coating of lauric acid and bovine serum albumin (SEON^LA-BSA^) were synthesized as previously described [11]. Briefly, iron oxide nanoparticles were synthesized under Argon atmosphere at 80 °C using alkaline co-precipitation. They were subsequently coated *in situ* with lauric acid and purified by dialysis (MWCO 8000 Da). The so-prepared SEON^LA^ were then additionally coated with albumin using excess bovine serum albumin (BSA) solution (6 wt % BSA solution in water).

Excess protein was then removed using a Spectrum Labs KrosFlo^®^ Research II ultrafiltration unit (SpectrumLabs, Los Angeles, CA, USA) with a tangential filter MWCO of 100 kDa. Before use, the filter was conditioned with ultrapure water for 15 min at a constant flow rate of 5 mL/min. Using constant feed flows of 15 mL/min, the transmembrane pressure was fixed to 0.50 bar by applying pressure to the tube after the filter. Ultrapure water was used as the washing agent. By determining the weight of the permeate, we were able to precisely control the washing process. The total iron concentration of all samples was determined using a UV-VIS spectroscopic method [32]. Briefly, 20 µL of the respective suspension were dissolved in 980 µL of hydrochloric acid 25% at 95 °C for 30 min. Fifty-microliter aliquots of this solution were then mixed with 80 µL of hydroxyl ammonium chloride solution 10% and 500 µL sulfosalicylic acid solution (20%). Five hundred microliters of ammonia solution 25% were added to change the pH, and the volume of the solution was adjusted to defined volumes. The extinction was measured at 405 nm, and the iron content of this lysate was determined using iron solutions in concentrations ranging from 1.5 to 15 µg/mL as the external standard. In parallel, we also measured the iron concentration of concentrated SEON^LA-BSA^ with an MP-AES 4200 (Agilent, Santa Clara, CA, USA). Therefore, we dissolved 20 µL of the respective suspension in 980 µL of hydrochloric acid 25% at 95 °C for 30 min. We then diluted these solutions to 100 mL with ultrapure water. The total iron content was determined at an emission wavelength of 371.993 nm. External standards of iron solution in concentrations ranging from 0.01 to 2.5 µg/mL were used for calibration.

The filtration efficacy was then estimated by determining the drying loss of the sample. Six pre-weighed Eppendorf tubes were filled with 500 µL of the respective suspension and heated to 110 °C overnight until constant weight. From the differences of the remaining weight and the drying loss of SEON^LA^, we were able to calculate the total amount of remaining albumin in the SEON^LA-BSA^ sample. Freundlich and Langmuir isotherms were calculated from the equilibrium adsorption *q*_E_ at defined albumin concentrations c in solution. The linear plots were displayed using the well-known formula for the Langmuir isotherm,

(1) $$\frac{1}{q_{E}}=\frac{1}{q_{max}}+\;\frac{K_{L}}{q_{max}}\times \;\frac{1}{c}$$

where *K_L_* is the Langmuir sorption coefficient, *q_E_* is the equilibrium adsorption. *q*_max_ is the maximum adsorption of the sorbent, while *c* stands for the equilibrium concentration. The Freundlich model is described by:

(2) $$\text{log}\left(q_{E}\right)=logK_{F}+\;\frac{1}{n}\text{log}(c)$$

where *K_F_* is the Freundlich sorption coefficient, *q_E_* is the equilibrium adsorption and *c* the equilibrium concentration.

The KrosFlo unit was also used to concentrate the samples. Using the same filters as in the filtration experiments, SEON^LA-BSA^ suspensions were concentrated up to 4-fold (*v*/*v*).

### 3.3. PCCS and Zeta Potential Measurements

Size measurements were performed with a Nanophox (Sympatec, Clausthal-Zellerfeld, Germany) photon cross-correlation spectrometer. The respective suspensions were diluted to a total iron concentration of 25 µg/mL with ultrapure water or RPMI 1640 cell culture medium and measured in triplicate at 25 °C. Auto non-negative least sqare analysis (Auto-NNLS) fit was used to obtain the size distributions.

pH-dependent electrophoretic mobility and size measurements enable a direct surface characterization of lyophobic colloids [21]. For pH-dependent hydrodynamic size and electrokinetic mobility (zeta potential) measurements, particles before and after filtration with a two-fold excess of ultrapure water were diluted to a total iron concentration of 25 µg/mL in 10 mM sodium chloride solution. The pH was adjusted to values from 3 to 10 using either hydrochloric acid or sodium hydroxide solution. Zeta potentials and hydrodynamic sizes were determined after 30 s of sonication using a Malvern Zetasizer (Malvern Instruments, Worcestershire, UK). All measurements were performed in triplicate at 25 °C.

### 3.4. Vibrating Sample Magnetometry

The magnetic properties of the samples were characterized using a vibrating sample magnetometer (Model 7407, Lake Shore-Cryotronix Inc., Westerville, OH, USA). The magnetization curves of the samples were measured for an increasing, as well as for a decreasing magnetic field with maximum field strength in the range of 1000 kA/m. The saturation magnetization M_S_ of the fluids was then calculated by plotting M against 1/H, enabling an extrapolation for external magnet fields H tending to infinity.

### 3.5. Blood Stability Assays

Freshly-extracted and EDTA-stabilized human blood was used to investigate the blood stability of the particles. The respective samples were diluted 1:2 (*v*/*v*) in whole blood. After 15 min of incubation, 1 µL of the respective sample was placed on a glass slide and investigated with a Zeiss Axio observer Z1 microscope (Zeiss Optics, Jena, Germany). SEON^LA^ and PBS were used as the positive and the negative control.

### 3.6. Cellular Uptake Assays

The cellular uptake of different concentrations of filtered and unfiltered SEON^LA-BSA^ into Jurkat cells, a human T-lymphoma cell line, was investigated using a method adapted from Dadaschzadeh *et al.* [24]. For experiments, 3.2 × 10^5^ Jurkat cells were seeded into 25 cm^2^ cell culture plates. After 24 h, SEON^LA-BSA^ were added to the cell culture media with a final concentration of 0, 20, 100 and 200 mgFe/mL, which correspond to 0-, 4.8-, 24- and 48-mgFe/cm^2^ cell culture plate areas (constant concentration of SPIONs in the cell culture media and on the plate surface area throughout all experiments). Cells were incubated another 48 h. After incubation, cell numbers and viability were determined with the MUSE^®^ Cell Analyzer (Merck-Millipore, Billerica, MA, USA). After treatment with SEON^LA-BSA^ and harvesting of the cells as described above, defined cell numbers (4 × 10^5^) were collected by centrifugation (5 min, 1000× *g*, 4 °C) and washed with PBS. The cell pellet was resuspended in 100 µL 10% sodium dodecyl sulfate and stored at −20 °C until further processing. After thawing, the cell lysates were agitated (300× *g*) for 5 min at 95 °C and immediately vortexed at high power to crop genomic DNA. Afterwards, the cell lysates were incubated for 1 h in an ultrasonic bath to ensure a homogeneous suspension of the SPIONs within the cell lysate. Un-filtrated SEON^LA-BSA^ standards (0, 0.5, 1.0, 1.5, 2.0, 5.0, 10, 15, 20 and 50 µgFe/mL) were prepared in 10% SDS and in non-treated cell lysate solutions. The optical density of the different suspensions was then measured at 370 nm (OD370). In detail, 50-µL cell lysates or standards were pipetted into 96-well plates (Techno Plastic Products AG, Trasadingen, Switzerland), and the OD_370_ was measured in a spectrophotometer (FilterMax F5, Molecular Devices, Sunnyvale, CA, USA). Standard dilutions of SPIONs enable quantification of iron oxide nanoparticle concentration in cell lysates. The reliability of this colorimetric assay is comparable to other methods like flow cytometry, magnetic particle spectroscopy (MPS) or atomic absorption spectroscopy (AAS) [33]. As the amount of cells was quantified before cell lysis, the SPION concentration was subsequently normalized to the cell number.

Statistical analyses were performed using a Student’s *t*-test in MS Excel.

### 3.7. SAR and T_max_ Determination

SAR values were determined by the mass normalized temperature increase at initial times after the onset of the alternating magnetic field (AMF, H = 15.4 kA/m, f = 435 kHz) in the linear range of the temperature curve [25,34]. For all SAR measurements, 200 µL of the appropriate SEON^LA-BSA^ concentration (original SPION concentrations diluted 1:2 in H_2_O) were transferred into FACS tubes (5 mL polystyrene round-bottom tube, BD Bioscience, Franklin Lakes, NJ, USA), and a fiber optic temperature sensor (TS5, Optocon AG, Dresden, Germany) was inserted centrally into the fluid. To minimize external influences during magnetic heating (e.g., caused by the cooling system of the coil) and to allow comparability among different experiments, samples were placed in an insulator before starting the measurement. By using the same experimental setup (including the used materials) for all measurements and determining the temperature in a closed system directly in a central position of the ferrofluid, thermal losses were minimized. SAR calculations were performed using the following equation:

(3) $$\text{SAR}=c\times \frac{m_{F}}{m_{P}}\times \frac{∆T}{∆t}$$

(*c*: specific heat capacity of the sample, *m_P_*: mass of SPION, *m_F_*: mass of fluid, *∆T/∆t*: maximum value of the linear slope at initial times).

Additionally to the SAR measurements, the highest achievable temperature (*T*_max_) of the SEON^LA-BSA^ suspension was determined at the saturation phase of the recorded temperature curve. For all measurements, H_2_O was used as the negative control.

## 4. Conclusions

Tangential ultrafiltration provides a highly-efficient alternative for the removal of macromolecules from ferrofluid suspensions. Only free albumin is removed from SEON^LA-BSA^, as neither the surface properties nor the hydrodynamic sizes change by filtration. This is further supportive of the formation of a core-shell structure of albumin on the surface of lauric acid-coated SPIONs, which was proposed earlier [11]. Cellular uptake is only moderately affected, as the particles have slightly reduced uptake after filtration. SEON^LA-BSA^ can be concentrated up to four-fold by tangential ultrafiltration without compromising colloidal stability in biorelevant media. The magnetic properties of the suspension as a whole are thereby greatly enhanced, and the saturation magnetization increases up to 1667.9 A/m in an iron concentration-dependent manner. This also enables higher maximum temperatures in magnetic hyperthermia using the same volume of ferrofluid. The therapeutic benefit of these temperatures, as well as the effect of the higher saturation magnetization on *in vivo* biodistribution during magnetic targeting are still to be investigated and shall be a topic of our future work. The presented results conclusively demonstrate the applicability of tangential flow ultrafiltration for the purification and concentration of macromolecule-coated SPIONs for an optimization of magnetic treatment.

## Supplementary Materials

Supplementary materials can be found at http://www.mdpi.com/1422-0067/16/08/19291/s1.
