# Supplementary material for: Tangential Flow Ultrafiltration Allows Purification and Concentration of Lauric Acid-/Albumin-Coated Particles for Improved Magnetic Treatment
Source: Int J Mol Sci. 2015 Aug 14;16(8):19291–307. doi: 10.3390/ijms160819291 (PMC4581297; doi:10.3390/ijms160819291)
Supplement: Supplementary file 1 [file ijms-16-19291-s001.pdf]

Supplementary Information

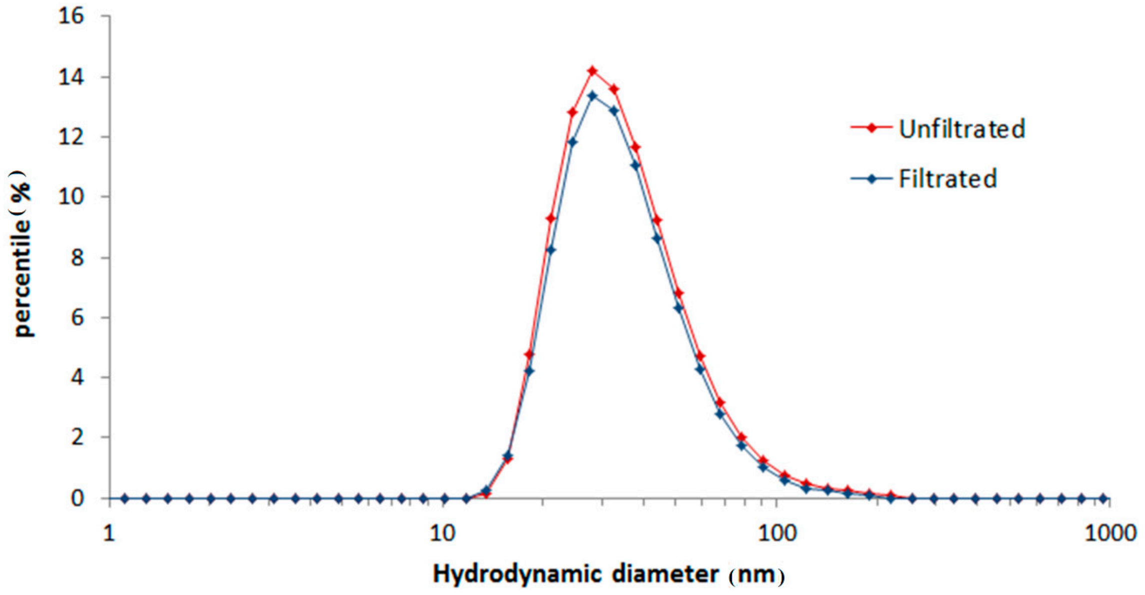

Figure S1. Size distribution of SEON$^{LA\text{-}BSA}$ at pH 7 before (red dots) and after (blue dots) filtration. Both samples are displayed as distribution by volume. The dots represent the mean of n = 3 individual measurements. The lines are drawn to guide the eye.

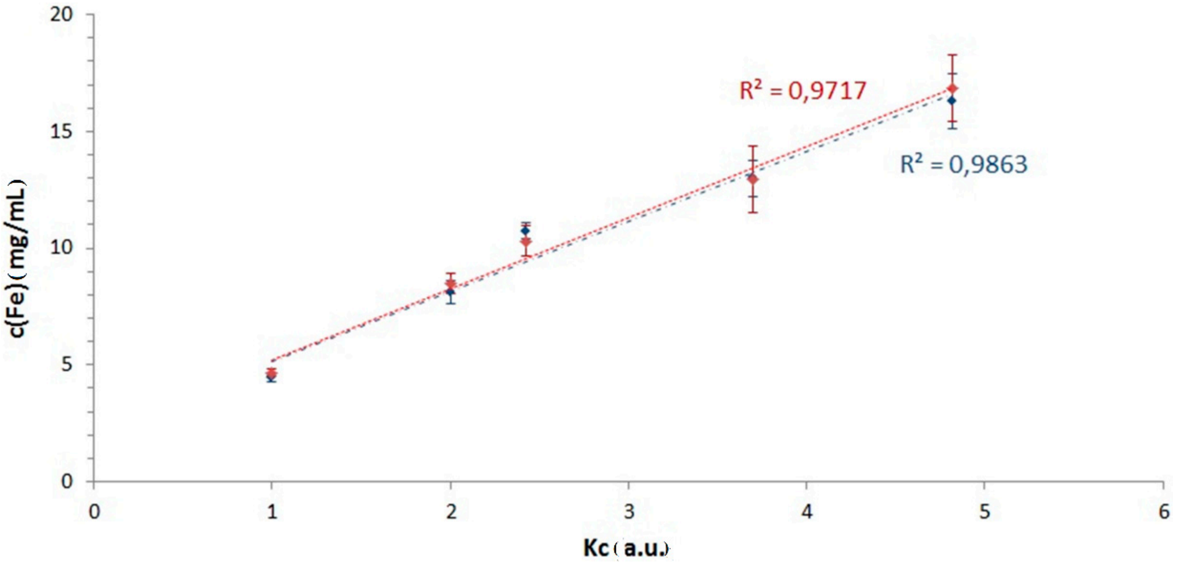

Figure S2. Comparison of iron content measurements with UV-VIS and MP-AES. SEON$^{LA\text{-}BSA}$ samples with different concentration factors K_C were measured by UV-VIS (Red dots and regression line) and MP-AES (Blue dots and regression line). All measurements were performed in triplicates. The values are displayed as mean ± standard deviation.
